# Immunotherapy in Basal Cell Carcinoma

**DOI:** 10.3390/jcm13195730

**Published:** 2024-09-26

**Authors:** Loredana Ungureanu, Alina Florentina Vasilovici, Salomea-Ruth Halmágyi, Ioana Irina Trufin, Adina Patricia Apostu, Manuela Prisecaru, Simona Corina Șenilă

**Affiliations:** 1 Department of Dermatology, “Iuliu Hațieganu” University of Medicine and Pharmacy, 400006 Cluj-Napoca, Romania; loredana.ungureanu@umfcluj.ro (L.U.);; 2 Department of Dermatology, Emergency County Hospital, 400006 Cluj-Napoca, Romania; 3 Clinical Hospital of Infectious Diseases, 400003 Cluj-Napoca, Romania

**Keywords:** basal cell carcinoma, imiquimod, immunotherapy, check point inhibitors

## Abstract

Basal cell carcinoma (BCC) is the most frequent of all cancers, with an increasing incidence. The first line therapy is surgical excision, but topical therapies can be used in low-risk superficial BCCs, while the more advanced, unresectable, or metastatic BCCs benefit from systemic therapies with hedgehog inhibitors and immunotherapy. The purpose of this review is to highlight local and systemic immunotherapies and their efficacy in the management of BCCs. Local therapies can be considered in superficial and low-risk nodular BCCs, with imiquimod frequently used for its antitumor and immunoregulatory properties. Imiquimod alone demonstrated higher histological clearance rates, but patients treated with imiquimod experienced more adverse events than ones treated with other therapies. Imiquimod can be used as an adjuvant before Mohs micrographic surgery and can also be combined with other local therapies, like curettage, electrodesiccation, cryosurgery, and photodynamic therapy, with some treatment methods yielding results comparable with the surgery. Interferons and Interleukin-2 were evaluated in a small number of studies with different results. Systemic immunotherapies with programmed death-ligand 1 (PD-L1) inhibitors showed inconsistent results in patients with advanced BCCs, being effective in some patients that progressed on or were intolerant to hedgehog pathway inhibitors (HHI).

## 1. Introduction

Basal cell carcinoma (BCC) is the most prevalent form of cancer among Caucasians, with a rapidly rising incidence. It is estimated that BCC represents roughly 50% of all cancer cases in the United States, although the exact number of cases remains uncertain [1,2]. Although associated with a very low metastatic rate (<0.1%), BCC can produce substantial local destruction and disfigurement, as it is able to invade soft tissue, cartilage, and bone [1,2]. Moreover, despite appropriate treatment, BCC is associated with a considerable risk of recurrence, the risk being highest in the first year after surgical removal [2,3]. Furthermore, Schreiber et al. demonstrated that within the first year following treatment, patients with a history of a single BCC developed one, two, or three new BCCs in 36%, 16%, and 8% of cases, respectively [3]. Metastases may affect regional lymph nodes, lungs, bone marrow, and bones [3].

BCC development is an interplay of intrinsic and extrinsic risk factors [4]. Several phenotypic features, especially light complexion and fair Fitzpatrick phototypes, are associated with a higher risk of BCC. Phototypes I–III are independent risk factors for BCC development compared with phototypes IV–VI. Individuals with blonde and light blonde to red hair, as well as those with light blue or green eyes have an increased risk for BCC. Freckles in childhood are another phenotypic factor leading to BCC [4]. Genome-wide association studies have identified several loci linked to an increased susceptibility to BCC. Mutations in PTCH1, PTCH2, SUFU, and smoothed genes, as well as the existence of MC1R variants, are associated with BCC [4]. The major environmental risk factor for BCC development is represented by solar UV radiation, with both intermittent and chronic sun exposure increasing the risk. The use of indoor tanning devices also poses a risk, especially in young females. Radiotherapy and alcohol consumption are other known external BCC risk factors [4]. A significant association between BCC and immunosuppression has been documented in cases of both iatrogenic and non-iatrogenic immunosuppression [4].

Recently, the European Association of Dermato-Oncology proposed a new classification for BCCs into “easy to treat” and “difficult to treat” [5]. The first line treatment is represented by surgical excision, either standard or micrographically controlled surgery, with the last being recommended for high risk, recurrent BCC as well as BCCs located on critical anatomical sites. In low-risk, superficial BCCs, topical and destructive therapies can be used. According to guidelines, a multidisciplinary tumor board should decide the management in “difficult to treat BCCs”, with treatment options being represented by hedgehog inhibitors, immunotherapy, radiotherapy, electrochemotherapy, and surgery [5].

The present review aims to discuss the role of immunotherapy—both local and systemic—in the management of BCC.

## 2. The Immune Microenvironment of Basal Cell Carcinoma

**Figure 1:**
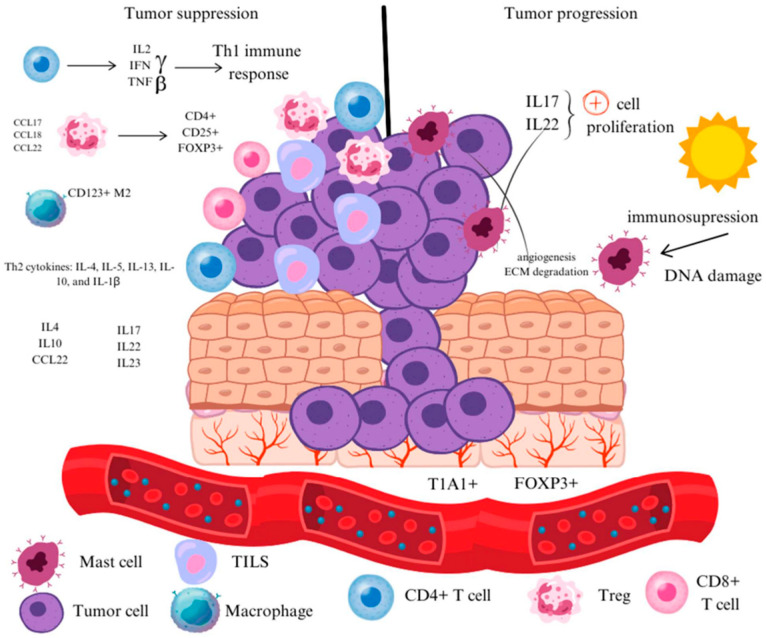
The immune microenvironment of basal cell carcinoma (TILS—tumor infiltrating lymphocytes; IL2—interleukin-2; IL17—interleukin-17; IL22—interleukin-22; IL4—interleukin-4; IL10—interleukin-10; IL5—interleukin-5; IL13—interleukin-13; IL10—interleukin-10; IL1β—interleukin 1β; IFN γ—Interferon gamma; TNF β—Tumor Necrosis Factor β; Treg—Regulatory T cells; Th1 immune response—Type 1 T Helper immune response; ECM—extracellular matrix; Th2—Type 2 T Helper).

The immune system plays a critical role in both the suppression and progression of human cancers, including basal cell carcinoma (BCC). The primary environmental risk factor for BCC development is exposure to ultraviolet radiation (UVR), which induces DNA damage and creates an immunosuppressive environment that facilitates tumor progression [6]. Immunosuppression is another major risk factor; increased rates of BCC are documented among patients undergoing long-term immunosuppressive therapy as well as in those with non-iatrogenic immune deficiencies [7]. The microenvironment in which basal cell carcinoma (BCC) is initiated and progresses consists of a diverse array of cells, extracellular matrix components, and signaling molecules, all of which engage in complex and dynamic interactions [8,9] (Figure 1).

Multiple studies showed a predominantly T cell infiltrate in BCC, with the tumor infiltrating lymphocytes (TILS) able to promote or inhibit tumor survival [10,11,12,13,14,15]. The presence of TILS in BCC suggests that the host initiates an immune response that could control tumor development since BCC is an immunogenic tumor. Moreover, in a small proportion of BCCs, partial or complete regression has been described in histopathological reports [6,16].

Histologic regression in BCC is divided into active and previous regression. Active regression is marked by a lymphocytic infiltrate that penetrates and surrounds the tumor nest, disrupting the regular tissue architecture. In contrast, previous regression is characterized by the presence of newly formed eosinophilic collagen in the dermis. This is accompanied by a reduction in skin appendages within the scarred area, an increased number of blood vessels, and a variable infiltrate of lymphocytes and plasma cells [17].

Hunt et al. analyzed 45 primary BCCs and observed a significantly higher number of CD3+ and CD4+ T cells infiltrating regressing tumors compared to non-regressing ones. They also found increased expression of the interleukin-2 receptor in the regressing tumors [17]. Wong et al. reported a significant increase in IFNγ levels as well as a trend toward elevated IL2 and TNFβ in actively regressing BCCs compared to non-regressing cases. This suggests the induction of a Th1 immune response against the tumor, primarily involving activated macrophages and cell-mediated immunity [18]. Fujimara et al., using immunohistochemical staining, examined the infiltrating cells in regressing tumor areas. They found few Foxp3+ cells but numerous T1A1+ cells infiltrating the dermis. Although immunosuppressive CD163+ M2 macrophages and MMP9+ cells were detected, these cells were predominantly located around the remaining tumor cells rather than infiltrating the tumor like cytotoxic T cells [19].

Kaporis et al. compared the genomic, protein, and cellular microenvironment of BCC with that of normal skin. Their study revealed that phenotypic Tregs (CD4+ CD25+ FoxP3+) surround epithelial aggregates of BCC. They also found that BCC-associated myeloid dendritic cells were primarily immature. Additionally, there was an increased expression of IL4, IL10, and CCL22 promoting a Th2 environment, along with elevated expression of interferon-associated genes (IFI27, IRF1, IRF7, and G1P2) supporting a Th1 response. The findings suggest that the immune microenvironment of BCC is in a dynamic state, characterized by a weakened immune response and a partial host antitumor response [20].

Omland et al. compared the T-reg density of facial BCC with peritumoral skin and non-ultraviolet exposed skin from the buttocks. Their study showed that T-regs are attracted to and accumulated in BCC, with T-regs found at a higher concentration in the peritumoral skin. In contrast, in the non-UV-exposed skin, no T-reg expression was found, indicating the important role of T-regs in the formation of an immunosuppressed niche in facial skin with pathogenetic consequences for the development of skin cancer [20]. They also found a high expression of the chemokines CCL 17, CCL 18, and CCL 22, involved in T-reg attraction, both in BCC and in peritumoral skin [15].

Beksac et al. conducted a study to examine the effects of peritumoral immune infiltrates on BCC recurrence. They compared non-recurrent tumors with both primary and recurrent tumors from the recurrent group [21]. The study revealed an immunosuppressive tumor microenvironment in all three groups characterized by immune infiltrates rich in CD4+ helper T cells and M2 macrophages. No statistically significant difference was found in CD4 and CD8 expression in non-recurrent tumors. However, the primary tumors in the recurrent group exhibited significantly lower infiltration of CD8+ cytotoxic T cells compared to CD4+ T-helper cells [21]. Interestingly, in recurrent tumors, the median levels of CD4 and CD8 staining were similar. The authors concluded that a higher density of CD4+ T cells and a lower density of CD8+ cytotoxic T cells in primary tumors may lead to a weakened antitumor immune response, increasing the likelihood of BCC recurrence. In contrast, a stronger antitumor immune response, driven by a denser cytotoxic T-cell infiltrate, may occur in recurrent tumors [21].

Multiple studies have also identified a significant mast cell infiltrate in BCC, particularly at the tumor periphery. More aggressive BCCs are associated with higher numbers of mast cells [22,23,24,25]. Additionally, smoking, which is linked to increased peritumoral mast cell numbers, is also associated with more aggressive BCC variants. Mast cells are thought to promote UVB-induced immunosuppression, angiogenesis, and extracellular matrix degradation [6,26].

Studies indicate that BCC is characterized by elevated levels of Th2 cytokines, including IL4, IL5, IL13, IL10, and IL1β [6,27,28,29]. Furthermore, BCC exhibits higher levels of IL17, IL22, and IL23 compared to normal skin. In vitro exposure of BCC cell lines to IL17 and IL22 has been shown to promote cellular proliferation [30,31]. Although various studies report low levels of IFNγ in BCC, elevated IFNγ in the tumor infiltrate has been associated with tumor regression [18,30,32].

Programmed Cell Death Protein 1 (PD-1) plays a crucial role in inhibiting immune responses and promoting self-tolerance. It achieves this by modulating T-cell activity, inducing apoptosis of antigen-specific T cells, and preventing apoptosis of regulatory T cells [33]. Programmed Cell Death Ligand 1 (PD-L1) is a transmembrane protein that acts as a co-inhibitory factor in the immune response. PD-L1 binds to PD-1, reducing the proliferation of PD-1-positive cells, suppressing their cytokine secretion, and inducing their apoptosis [33]. The PD-1/PD-L1 axis is a key mechanism behind cancer immune evasion, allowing tumor cells to escape host immune surveillance [33]. Studies have shown that PD-L1 is upregulated on tumor cells and tumor-infiltrating lymphocytes (TILs), facilitating immune evasion [34].

Lipson et al. characterized PD-L1 and PD-1 expression patterns in the tumor microenvironment of 40 BCC specimens. They found that 22% of tumor cells and 82% of TILs and associated macrophages expressed PD-L1 [35]. In all cases, PD-1 expression was present on TILs, and 82% of cases with PD-L1 expression on infiltrating immune cells showed proximity to a PD-1-expressing cell [35].

Chang et al. investigated PD-L1 expression in both treatment-naïve and treated BCCs, including those recurring after surgery, radiotherapy, systemic chemotherapy, or topical therapy [34]. Among 138 BCC specimens from 62 patients, 89.9% showed PD-L1 expression in tumor cells, and 94.9% displayed PD-L1 expression in TILs. Notably, in a multivariable model adjusting for age, sex, and tumor location, PD-L1 staining intensity in tumor cells increased with the number of prior treatment modalities [34].

Gompertz-Mattar et al. evaluated PD-L1 expression and markers of local immune response in nodular, superficial, and morpheaform BCC. They compared these findings to normal, sun-exposed skin from the periphery of intradermal nevi [36]. BCC specimens exhibited higher PD-L1, CD8, and FOXP3 expression compared to sun-exposed skin. Interestingly, low-risk BCC subtypes (superficial and nodular) showed greater PD-L1 expression in both the intratumoral and stromal immune infiltrate compared to high-risk subtypes. Additionally, low-risk BCCs had more stromal lymphocytic infiltration, while high-risk BCCs exhibited higher stromal CD4 and CD8 expression [36].

## 3. Local Immunotherapy

### 3.1. Imiquimod

According to the EADO guidelines, topical therapies should be considered for patients with superficial or low-risk nodular BCC who decline surgical intervention or if surgery is contraindicated due to patient-related factors, such as age, comorbidities, concomitant medications, or logistical difficulties. Two agents are currently approved for this purpose: imiquimod 5% and 5% 5-fluorouracil (5-FU) [5].

Imiquimod is an immune response modifier that has been approved by the FDA for the treatment of genital and perianal warts, actinic keratosis, and superficial basal cell carcinoma. It stimulates both the innate and adaptive immune pathways and induces cytokine production, providing antiviral, antitumor, and immunoregulatory effects [37]. As a toll-like receptor (TLR)-7 and TLR-8 agonist, imiquimod triggers proinflammatory cytokine release through the activation of nuclear factor kappa-B (NF-kB). This activation leads to the production of various cytokines, including IFNα, TNFα, IL1, IL6, IL8, IL10, IL12 p40, granulocyte colony-stimulating factor, granulocyte/macrophage colony-stimulating factor, macrophage inflammatory protein, and macrophage chemotactic protein-1 [37,38,39,40]. The effects of imiquimod are also mediated by the recruitment and activation of plasmacytoid dendritic cells (PDCs) in the skin. It reduces adenylyl cyclase activity and indirectly stimulates the production of the Th1 cytokine IFNγ [37,41,42,43,44]. At higher concentrations, imiquimod induces apoptosis in tumor cells through the activation of Bcl-2 proteins and the caspase family of proteases [37,42]. Furthermore, imiquimod possesses anti-angiogenic properties. This is achieved through the production of anti-angiogenic cytokines, such as IFNs, IL10, and IL12, the upregulation of endogenous anti-angiogenic mediators, such as tissue inhibitors of matrix metalloproteinase, and the downregulation of pro-angiogenic molecules, including basic fibroblast growth factor and matrix metalloproteinase [37,45].

Jia et al. conducted a meta-analysis to assess the efficacy and safety of imiquimod compared to other treatments for patients with BCC [46]. They included 13 studies with a total of 4256 patients. Among these studies, six compared imiquimod to a vehicle, four compared it to MAL-PDT or fluorouracil cream, two compared it to surgery, and one compared it to radiotherapy. Imiquimod was found to have a significantly higher histological clearance rate (77.26%) compared to other treatments (4.54%). This higher clearance rate was observed for both superficial and nodular BCC when imiquimod was administered once daily, twice daily, or three times weekly [46]. In addition, imiquimod demonstrated better success rates, complete response rates, and tumor-free survival when compared to other treatments. However, the meta-analysis revealed that imiquimod was associated with a significantly higher incidence of adverse events (53.3%) compared to other treatments (35.8%). Specifically, patients treated with imiquimod experienced a higher incidence of itching, weeping, and headaches but a lower incidence of burning [46]. The authors concluded that imiquimod provides significant benefits in improving histological and composite clearance rates. Despite the higher rate of side effects, it could be considered a first-choice treatment for patients with BCC [46].

#### 3.1.1. Imiquimod and Surgical Excision

Vanaclocha et al. aimed to assess the cost-effectiveness of surgical excision compared to imiquimod 5% cream in the treatment of BCC. The effectiveness of surgical excision was 97% for dermatology services and 90% for non-dermatology services, while imiquimod (36 sachets) showed an efficacy rate of 82%. Savings per patient cured using topical imiquimod were EUR 55 in dermatology and EUR 375 in non-dermatology services. Though the study did not assess the risk of recurrence or the cost of treating failures, the authors concluded that imiquimod 5% cream is a cost-effective alternative to surgery for patients with superficial BCC [47].

The SINS trial, conducted by Bath-Hextall et al., compared excisional surgery to imiquimod for nodular or superficial BCC in low-risk sites, examining 3-year clinical clearance, cost-effectiveness, and cosmetic results. Imiquimod 5% cream was applied daily for 6 weeks for superficial BCC or 12 weeks for nodular BCC, while surgical excision was performed with a 4 mm safety margin. After 3 years, 84% of the imiquimod group was successfully treated, compared to 98% in the surgery group. Both groups had similar cosmetic outcomes. Adverse events were more common in the imiquimod group, but the surgery group experienced more adverse events during follow-up. The authors concluded that although surgery is superior, imiquimod may be a useful option for small, low-risk BCC [48].

Williams et al. provided 5-year follow-up data for the SINS study, using histopathology and health care records. Five-year success rates for imiquimod were 82.5%, compared to 97.7% for surgery, similar to three-year results. Most imiquimod treatment failures occurred within the first year, indicating long-term benefits for early responders. Despite surgery’s superior results, imiquimod is a viable alternative for low-risk BCC, with surgery reserved for cases unresponsive in the first year [49].

Tinelli et al. explored patients’ preferences for either surgery or imiquimod using a questionnaire from 183 SINS trial participants. The results showed that respondents generally preferred imiquimod over surgery, irrespective of their prior BCC experience. Concerns about cosmetic outcomes and side effects played a larger role in their preferences than clearance rates and costs [50].

Sinx et al. evaluated the treatment of nodular BCC using curettage followed by imiquimod versus surgical excision. Excision was performed with a 3 mm safety margin, while imiquimod cream was applied daily, 5 days a week, for 6 weeks after curettage. The 12-month recurrence-free rate was 86.3% for curettage and imiquimod and 100% for excision. However, the study could not establish curettage and imiquimod as non-inferior to excision. Clinically, the cosmetic outcome was rated better for curettage and imiquimod than for excision, particularly for BCC in the head and neck region [51]. In a discrete choice experiment conducted alongside the trial, Sinx et al. evaluated patient preferences between curettage with imiquimod and surgical excision for non-facial nodular BCC. Overall, 60% of patients chose surgery, while 40% opted for curettage with imiquimod. Patients who had experienced both treatments showed a nearly equal preference, with 49% choosing curettage with imiquimod and 51% selecting surgery [52].

De Macedo et al. explored the use of imiquimod as a neoadjuvant therapy followed by wide local excision in treating periocular nodular basal cell carcinoma (BCC). Their study reported a reduction in BCC recurrence after treatment with 5% imiquimod, with a three-year histological clearance rate of 100% for tumors smaller than 10 mm and 81.8% for those larger than 10 mm. For the larger lesions, surgery was performed after significant tumor reduction (one from 13.5 mm to 4.2 mm, and another from 27.2 mm to 7.6 mm), making reconstruction easier and reducing complications from wide excision. These findings highlight the neoadjuvant effect of imiquimod for patients with partial tumor clearance [53].

#### 3.1.2. Imiquimod and Mohs Surgery

Torres et al. conducted a study to evaluate the effectiveness of 5% imiquimod cream in treating BCC prior to excision by Mohs micrographic surgery. The study also aimed to determine if reflectance-mode confocal microscopy could help assess the need for surgical intervention following imiquimod treatment [54]. After biopsy confirmation, imiquimod or a vehicle was applied 5 times per week for either 2, 4, or 6 weeks. In the 6-week treatment group, confocal microscopy was used to assess the target tumor area at each visit and immediately before Mohs surgery. After excision, the samples were histologically evaluated and compared with confocal microscopy readings. The study showed that using 5% imiquimod cream before Mohs surgery significantly reduced the size of the target tumor. This resulted in smaller surgical defects compared to the vehicle group [54].

Thissen et al. assessed the possibility of treating superficial BCC left after incomplete Mohs surgery. In these cases, the aggressive and deep parts of the tumor were already removed [55]. Imiquimod 5% cream was applied three weeks after the defect was closed, covering at least 1 cm around the surgically treated area for six weeks. No recurrences were observed after a follow-up period of 20 to 34 months [55].

Buttler et al. investigated whether 5% imiquimod cream could reduce the number of Mohs surgery stages, defect size, costs, and the extent of reconstruction needed [56]. Patients applied imiquimod nightly for 6 weeks with occlusion, followed by a 4-week rest period before undergoing Mohs surgery. The study found that imiquimod did not reduce the number of Mohs stages, defect size, or the cost of the procedure and repair. This study, however, had a smaller sample size than others. The authors noted lower-than-expected tumor clearance in the imiquimod group, particularly with nasal nodular BCC, which proved more resistant to treatment than BCCs at other sites. They strongly recommend histological assessment of nasal BCCs treated with imiquimod [56].

Van der Geer et al. conducted a study on 70 patients to assess whether pretreatment with 5% imiquimod cream could reduce tumor size, surgical defect size, the number of Mohs stages, and reconstruction time [57]. In this study, the imiquimod group applied the cream for 4 weeks before surgery, while the control group underwent Mohs surgery alone. The results showed that pretreatment with imiquimod significantly reduced tumor and surgical defect size in primary nodular BCCs on the face. Additionally, the study observed fewer Mohs stages and significantly shorter reconstruction times in the imiquimod group [57].

#### 3.1.3. Imiquimod and Curettage and Electrodesiccation (C&D)

C&D is a common treatment for nodular BCC. However, residual tumors are found immediately after treatment in 20% to 40% of cases. Spencer et al. conducted a study to investigate whether the administration of imiquimod after C&D improves treatment outcomes [58]. In this study, 20 patients underwent 3 cycles of C&D followed by the application of imiquimod 5% cream or vehicle cream once daily for 1 month. The results showed that imiquimod 5% cream significantly reduced the frequency of residual tumors and improved cosmetic appearance. Most scars in the control group were atrophic and hypopigmented, while in the imiquimod group, the scars were flat and slightly pink [58].

In another study, Wu et al. evaluated the efficacy and cosmetic outcome of curettage followed by imiquimod 5% cream in the treatment of primary nodular BCC located on the trunk and limbs. Curettage was used to de-bulk the lesion and confirm the histopathological diagnosis. Aggressive subtypes, such as micronodular and morpheaform BCC, were excluded. After curettage, imiquimod cream was applied daily for 6 to 10 weeks. Three months post-treatment, all lesions were excised. The study found that 94% of treated lesions were histologically clear of BCC, with overall favorable patient-reported cosmetic outcomes [59].

Rigel et al. assessed the efficacy of curettage without electrodesiccation, followed by imiquimod 5% cream, applied 5 times weekly for 6 weeks [60]. No clinical recurrences were observed after a one-year follow-up, and cosmetic outcomes were rated as very good to excellent. Tillman Jr et al. replicated this protocol in a study involving 101 tumors. The clearance rate was 96% after an average follow-up of 36 months. This treatment regimen was well tolerated and produced favorable cosmetic results [61].

Ondo et al. retrospectively evaluated patients with superficial BCC treated with curettage followed by topical imiquimod 5% cream (C&I). The control group consisted of patients treated with other methods [62]. In the C&I group, the tumor was curetted with a 2–4 mm peritumoral margin, followed by the application of imiquimod 5% cream daily to the lesion and surrounding skin. The treatment continued until a typical response (erythema, crusting, superficial erosion) was observed. C&I for superficial BCC was well tolerated, requiring an average of only 19 days of imiquimod application to achieve a response. The study reported a high recurrence-free survival at 10 years (97.5%) compared to 84.1% in the control group. Lower recurrence-free survival rates were noted on the nose (84.4%) and scalp (86.9%) [62].

#### 3.1.4. Imiquimod and Cryosurgery

Gaitanis et al. conducted multiple studies to evaluate the combination of cryosurgery with daily imiquimod application, a treatment termed immunocryosurgery, for BCC [63,64,65,66,67,68,69,70]. This protocol typically involves a standard 5-week cycle. Imiquimod 5% cream is applied nightly over the lesion and a 0.5 cm margin of surrounding skin. On day 14, cryosurgery is performed, involving two freeze-thaw cycles of 10–20 s each, targeting the tumor and a 0.5–1.0 cm margin around it. For larger BCCs, the cycle can be extended, with additional cryosurgery sessions performed every 2–3 weeks if necessary [71]. The authors noted that immunocryosurgery’s main disadvantage is the local discomfort caused by the inflammatory response. After the cryosurgery session, particularly for large lesions, patients may experience flu-like symptoms, such as low-grade fever, loss of appetite, and fatigue. The advantages of immunocryosurgery from a health system perspective include its low cost, ease of outpatient application, and minimal training required for physicians. For patients, the treatment offers high tumor control efficacy with excellent aesthetic and functional outcomes [63,64,65,66,67,68,69,70,71].

For BCCs with a diameter of up to 2 cm, the efficacy of immunocryosurgery is comparable to first-line surgical therapy, with a clearance rate of 97.5% [65]. If relapse occurs after one cycle, a second 5-week cycle results in a 99% clearance rate for tumors up to 2 cm in diameter [68]. The authors highlight that immunocryosurgery is significantly more effective than imiquimod monotherapy for nodular BCC or cryosurgery alone. Tumors typically requiring Mohs surgery, such as those with undefined borders or located in high-risk areas, are not contraindications for immunocryosurgery [65,68,71].

#### 3.1.5. Imiquimod and Photodynamic Therapy (PDT)

Osiecka et al. conducted a study to evaluate the effectiveness of photodynamic therapy (PDT) combined with imiquimod in treating recurrent BCC. A total of 34 patients with histopathologically confirmed BCC participated in the study. Ten patients received Levulan-PDT combined with a placebo cream (Eucerin), while 24 patients were treated with Levulan-PDT and imiquimod. Photodynamic diagnosis was used to identify suspicious cancer foci. In the group treated with PDT and placebo, 60% experienced complete tumor disappearance, and 40% showed a significant reduction in tumor size. In contrast, 75% of patients treated with both PDT and imiquimod achieved tumor clearance, while 25% experienced significant tumor reduction. Both treatment groups showed minimal scarring and favorable cosmetic outcomes, with a higher clearance rate observed in the group receiving dual therapy with imiquimod [72].

While surgical excision remains the first-line treatment for BCC and often achieves higher clearance rates, imiquimod has been demonstrated to be an effective alternative, particularly in less invasive and cost-effective contexts. It can be used as monotherapy or in combination with other treatments. Various dosing regimens have been explored, typically depending on factors such as BCC subtype, lesion location, and the preferences of both physicians and patients. However, variability in treatment responses across studies suggests that imiquimod’s efficacy may be influenced by lesion location, warranting further research in this area [73].

### 3.2. Interferons

Interferons (IFNs) are naturally occurring glycoproteins secreted by cells in response to viral infections, as well as synthetic and biological inducers. They have a wide range of biological activities, including antiviral, antiproliferative, and immunomodulatory effects. These properties have been demonstrated in multiple in vitro and in vivo studies. IFNs are classified into three types: Type I includes IFN-*α*, IFN-*β*, IFN-*ε*, IFN-*κ*, and IFN-*ω*; Type II consists of IFN-*γ*; and Type III includes IFN-*λ* [74].

The antitumor activity of interferons is primarily mediated through the activation of natural killer (NK) cells and macrophages, the enhancement of lymphocyte cytotoxicity, and the upregulation of major histocompatibility antigens. In basal cell carcinoma (BCC), exposure to Type I interferons induces the expression of the CD95 receptor, which triggers programmed cell death [75].

Several interferons, such as INF-alpha-2a, INF-alpha-2b, and recombinant INF-beta-1a, are commercially available. These are currently used off-label for treating both superficial and nodular BCC. Various dosing regimens have been employed, with doses ranging from 1.5 to 30 million units administered 1 to 3 times per week for 3 to 6 weeks. Efficacy rates for INF-alpha range from 52% to 98%, while recombinant IFN-beta-1 showed a response rate of 67% in one study [74,75,76,77,78].

Despite the favorable cosmetic outcomes seen in most studies, interferon-based treatments often require multiple sessions to achieve satisfactory responses. This can make the treatment costly and may lead to systemic side effects, which can include flu-like symptoms such as fever, headache, muscle pain, and nausea. These side effects are dose dependent, making the treatment less tolerable than other topical or intralesional therapies [74,75].

Given the lower efficacy of interferon therapy compared to other approved treatments, as well as its dose-dependent side effects and uncertain response in more aggressive subtypes, interferons should only be considered for patients who cannot undergo surgery. They may also be appropriate for treating BCCs located in anatomically challenging areas where surgery is difficult.

### 3.3. Interleukin-2 (IL-2)

Interleukin-2 (IL-2) is a cytokine produced by T cells that promotes the expansion of cytotoxic T cells, which are essential for targeting and eliminating tumor cells. Although systemic administration of IL-2 is associated with significant side effects and is poorly tolerated, intralesional administration of IL-2 can reduce these systemic adverse effects [76].

In an open-label, uncontrolled study, Kaplan et al. evaluated the efficacy and safety of perilesional PEG-IL-2 injections in patients with BCC. The study included 8 patients with 12 histologically confirmed primary BCCs. IL-2 was injected into the subcutaneous tissue around the lesion in a radial pattern. The study reported a clinical and histological cure rate of 66.6% (8 out of 12 lesions). Side effects were limited to local pain, swelling, and flu-like symptoms in one patient. Importantly, there were no significant changes in blood tests compared to baseline. The authors concluded that intralesional IL-2 warrants further investigation as a potential treatment for BCC [79] (Table S1).

## 4. Systemic Immunotherapy

Advanced BCC is rare but particularly challenging to treat. In less than 1% of patients, BCC can progress to locally advanced or metastatic stages, which carry a very poor prognosis. The median survival for metastatic BCC is 8 to 14 months, with a 5-year survival rate of just 10% [80].

Advanced BCC can be divided into two patient groups with distinct characteristics and response criteria. Locally advanced disease typically involves either a single very large tumor or multiple primary tumors that cannot be treated with surgery or radiotherapy with curative intent. In contrast, metastatic disease is characterized by locoregional or distant metastases [80]. Locally advanced BCC (laBCC) accounts for 0.8% of all BCC cases, while metastatic BCC (mBCC) has an estimated incidence of 0.0028–0.55%. However, the actual incidence of advanced BCC may be underestimated, as the registries of advanced BCC have been recently established [5].

BCC has a notably high tumor mutational burden, suggesting that it could be responsive to immunotherapies, such as programmed death-ligand 1 (PD-L1) inhibitors. Case reports have highlighted the potential of PD-L1 inhibitors to treat advanced BCCs, particularly in patients who are refractory to, experience recurrence after, or are intolerant to the treatment with hedgehog pathway inhibitors (HHIs) [1,2,5].

Immune checkpoint therapy with PD-1 antibodies has shown significant clinical value and strong therapeutic potential, notably improving progression-free survival and overall survival. Cancer treatment has now evolved beyond surgery, radiotherapy, chemotherapy, and targeted therapy, entering the era of immunotherapy. However, despite its remarkable effectiveness, immunotherapy is not without limitations, such as immune-related adverse events (irAEs), cytokine storms, and a relatively low response rate [81]. Many cancer patients either do not respond to immunotherapy, or their response is short-lived, with disease recurrence often seeming inevitable. This is largely due to the rapidly evolving resistance of the cancer cells [82]. Primary resistance to immunotherapy occurs when cancer does not respond to treatment that has never been used in the patient before, with the immune system failing to react, even with pharmacological stimulation. On the other hand, acquired resistance develops when a previously effective immunotherapy no longer works, despite having been successful in the past for that patient. To qualify as acquired resistance, at least six months of progression-free survival must be observed before the cancer progresses again.

Moreover, carefully balancing the efficacy and safety of treatment regimens is crucial and must be tailored to individual patient cases. Treatment-related adverse events associated with checkpoint inhibitors are numerous, with the most commonly reported adverse events including anemia (45.4%), fatigue (34.3%), and dysphagia (30%). Among grade 3 or higher adverse events, neutropenia (19.6%), hypertension (9.3%), and lymphopenia (10.3%) were the most frequent, along with pyrexia, diarrhea, skin toxicity, hematological events, thyroid dysfunction, endocrine disorders, and pneumonitis, among others. Although treatment-related death is rare, it can occur, often due to severe toxicity from combination therapies or aggressive immune responses, such as a cytokine storm [83].

### 4.1. Cemiplimab

Cemiplimab is a fully human monoclonal antibody of the IgG4 class that binds to the programmed cell death-1 (PD-1) receptor, blocking its interaction with PD-L1 and PD-L2 ligands. This blockade enhances anti-tumor T-cell responses. Cemiplimab is approved for treating adult patients with locally advanced basal cell carcinoma (laBCC) or metastatic BCC (mBCC) who have progressed on, or cannot tolerate, HHIs. The recommended dosage is 350 mg every 3 weeks, administered as a 30-min intravenous infusion [84].

Otsuka et al. demonstrated that HHI treatment can enhance adaptive immune responses by increasing MHC-I expression and promoting intra-tumoral infiltration of CD4+ and cytotoxic CD8+ T cells [85]. This may improve the likelihood of tumors responding to anti-PD-1 therapy [86]. Moreover, Chang et al. showed that PD-L1 expression of treated BCCs (treatment with HHI, platinum chemotherapy, gefitinib, topycal chemotherapy, surgery, and radiotherapy) was significantly increased in tumor cells and tumor-infiltrating lymphocytes compared with treatment-naïve BCCs (32% vs. 7%, respectively) [34].

Cemiplimab received regulatory approval based on a key phase 2, open-label, multicenter trial (NCT03132636) that investigated its use in patients with advanced BCC [87]. The trial enrolled 84 patients with laBCC, who had previously been treated with HHIs. These patients were not eligible for further HHI therapy due to disease progression (71%), stable disease after 9 months of HHI treatment (8%), or intolerance (38%). Patients received cemiplimab intravenously at a dosage of 350 mg every 3 weeks for up to 93 weeks or until disease progression or unacceptable toxicity occurred [87].

The trial’s primary endpoint was an objective response (OR), defined as the percentage of patients achieving a complete response (CR) or a partial response (PR). In the laBCC group, 31% of patients (26 individuals) achieved an OR, with 6% achieving a CR and 25% a PR. The median time to response was 4.3 months. Subgroup analyses indicated that the response was consistent across variables, such as age, sex, and previous HHI treatment outcomes. Cemiplimab was discontinued in 62% of patients, with the reasons being disease progression (35%), adverse events (16%), or patient decision (6%). The Kaplan–Meier estimate showed that 76% of patients were alive and without disease progression at 6 months, with 57% at 12 months. In terms of safety, grade 3–4 treatment-emergent adverse events (TEAEs) were reported in 40 patients (47.6%). The most common severe TEAEs were hypertension and colitis (4 patients, 4.8%), followed by fatigue, urinary tract infection, and visual impairment (3 patients, 3.6%). Importantly, there were no recorded grade 4 or grade 5 immune-related adverse events, and no treatment-related deaths occurred [87].

The same trial also enrolled a cohort of 54 individuals with metastatic disease. In the mBCC cohort, an OR was reported in 12 patients (22.2%), with 2 patients (3.7%) achieving a CR and 10 patients (18.5%) showing a PR, with an average time to response of 3.1 months. The Kaplan–Meier estimate indicated that 100% of patients were alive and free of disease progression at 6 months, and 58% at 12 months. Regarding safety, TEAEs of any grade occurred in 51 patients (94.4%), with the most common being fatigue (23 patients, 42.6%), diarrhea (20 patients, 37.0%), constipation (12 patients, 22.2%), and hypertension (12 patients, 22.2%). TEAEs of grade 3 or higher were reported in 23 patients (42.6%), with hypertension (6 patients, 11.1%) being the only one observed in more than 2 individuals. Four patients (7.4%) discontinued treatment due to adverse events. Serious TEAEs were reported in 16 patients (29.6%), and 2 patients died—one from staphylococcal pneumonia and another from hemoptysis—though neither death was attributed to cemiplimab [88].

Exploratory biomarker analyses in the cohort with laBCC showed no correlation between treatment response and baseline PD-L1 status, tumor mutational burden (TMB), or major histocompatibility class 1 (MHC-1) expression. Among 50 patients with evaluable PD-L1 expression, the OR was 26% in those with PD-L1 expression below 1% (n = 35) and 27% in those with PD-L1 expression of 1% or higher (n = 15), indicating that patients with low PD-L1 expression can still benefit clinically from cemiplimab [87].

### 4.2. Nivolumab

The UNICANCER AcSe NIVOLUMAB phase 2 basket trial (NCT03012581) evaluated nivolumab in 32 patients with advanced BCC, including 29 with laBCC and 3 with mBCC. Patients received 240 mg of nivolumab via IV infusion every 2 weeks for up to 24 months. The median follow-up period was 17 months (IQR 12-23). At the 12-week radiological evaluation, 22% of patients showed a response, with 3.1% achieving a complete response and 18.8% a partial response. Adverse events were reported in 28% of patients, with nearly half being treatment-related. Only one treatment-related adverse event led to discontinuation. The most frequent adverse events included diabetes mellitus, colitis, pneumonitis, myocardial infarction, lymphopenia, and bullous pemphigoid [89].

### 4.3. Pembrolizumab

In a proof-of-concept study by Chang et al., pembrolizumab was assessed as a monotherapy (200 mg IV every 3 weeks) or in combination with vismodegib for advanced BCC. The combination of pembrolizumab with vismodegib showed lower response rates compared to pembrolizumab alone, with objective response rates of 29% for the combination and 44% for pembrolizumab monotherapy [90].

Anti-PD-1 agents provide a therapeutic option for patients with advanced BCC who are not candidates for curative surgery or radiotherapy and have either progressed on or are intolerant to HHI. Clinical trials indicate that these immune checkpoint inhibitors can be effective for a subset of patients, though others may exhibit primary or secondary resistance to therapy.

## 5. Conclusions

BCC is the most prevalent cancer among Caucasians, and its incidence is increasing rapidly. The immune system plays a vital role in both inhibiting and promoting BCC progression. Surgery is usually the treatment of choice due to its high success rate and the ability to confirm complete tumor removal. However, topical and intralesional immunotherapies have proven effective and yield good cosmetic results. For patients with advanced BCC, including those with locally advanced or metastatic BCC that cannot be treated with curative surgery or radiotherapy, HHI are approved as first-line therapy. Anti-PD-1 agents provide an alternative treatment for patients with advanced BCC who have either progressed on or cannot tolerate HHI. Although immunotherapy, both local and systemic, can result in durable responses in some patients, many may not respond or may eventually lose their response. The identification and use of biomarkers to predict and treat patients more likely to respond is a promising area of ongoing research focused on personalized treatment.

## Data Availability

No new data were created.

## Supplementary Materials

The following supporting information can be downloaded at https://www.mdpi.com/article/10.3390/jcm13195730/s1, Table S1: Local immunotherapy in basal cell carcinoma (BCC).
