# Supplementary material for: Immunotherapy in Basal Cell Carcinoma
Source: J Clin Med. 2024 Sep 26;13(19):5730. doi: 10.3390/jcm13195730 (PMC11476842; doi:10.3390/jcm13195730)
Supplement: Supplementary file 1 [file jcm-13-05730-s001.zip › jcm-3214119-supplementary.pdf]

Local immunotherapy	Patients' suitability	Side effects	Relapse rates
Imiquimod monotherapy [47]	▪ Superficial BCC<br>▪ Nodular BCC	▪ itching<br>▪ weeping<br>▪ headache<br>▪ erythema<br>▪ flu-type symptoms	14% before 1 year,<br>1% at 3 years [49]
Curettage +Imiquimod	▪ Nodular BCC<br>▪ Superificial BCC	▪ Erythema<br>▪ Crusting<br>▪ Superifical erosion	13.7% before 1 year [52]<br>4% at 3 years [61]<br>2.5% at 10 years [62]
Imiquimod +Mohs surgery [73]	▪ Adjuvant in superficial BCC [55]<br>▪ Neoadjuvant in nodular BCC [57]	▪ local erythema<br>▪ itching<br>▪ crusting<br>▪ irritation	0% at 2-3 years [55]<br>0% at 19 months [57]
Imiquimod+cryosurgery (imunocryosurgery)	▪ Nodular BCC<br>▪ BCC with indication for Mohs surgery (undefined borders, prone to relapse) [65,68,71]	▪ local discomfort<br>▪ flu-like symptoms	3% at 5 years [65]
Imiquimod and PDT	▪ Recurrent BCC [72]	▪ erythema<br>▪ painful edema<br>▪ burning sensation<br>▪ itching<br>▪ erosions	8.3% at 10-15 months [72]
Interferons	▪ High-risk facial BCC [78]	▪ moderate to severe flu-like symptoms<br>▪ nausea<br>▪ local pain	12.3% at 55 months [78]
IL-2	▪ Not reported	▪ local pain<br>▪ edema<br>▪ erythema<br>▪ flu-like symptoms	Not reported

Table S1. Local immunotherapy in basal cell carcinoma (BCC)

BCC – basal cell carcinoma, IL-2 – Interleukin-2, PDT – photodynamic therapy
